# Effects of silver diamine fluoride on oral bacteriome and mycobiome: a randomized clinical trial

**DOI:** 10.1186/s12903-025-06986-0

**Published:** 2025-10-21

**Authors:** Mayura Manerkar, Vivianne Cruz de Jesus, Betty-Anne Mittermuller, Victor H. K. Lee, Sarbjeet Singh, Mary Bertone, Prashen Chelikani, Robert J. Schroth

**Affiliations:** 1 Dr. Gerald Niznick College of Dentistry, Rady Faculty of Health Sciences, University of Manitoba, Winnipeg, MB Canada; 2 Children’s Hospital Research Institute of Manitoba, Winnipeg, MB Canada; 3 Shared Health Inc, 507-715 McDermot Avenue, Winnipeg, MB R3E 3P4 Canada

**Keywords:** Bacteria, Children, Early childhood caries, Fungi, Microbiome

## Abstract

**Background:**

Silver diamine fluoride (SDF) is a simple and non-invasive agent used to arrest early childhood caries (ECC). This study aimed to investigate potential changes to the oral microbiome in children with ECC who were treated with SDF and sodium fluoride (NaF) varnish at three different frequency regimens.

**Methods:**

Forty-five children (*n* = 15 per group) with ECC were recruited from community-based dental clinics in Winnipeg, Canada into an open-label, parallel-group, randomized clinical trial testing three different treatment frequency regimens of SDF. A total of 195 carious lesions were treated with two applications of 38% SDF and 5% NaF varnish (and assessed over three study visits one month, four months, or six months apart. Dental plaque samples were collected at each visit. Sequencing of the V4-16 S rRNA and ITS1 rRNA genes were used to study the supragingival plaque microbiome.

**Results:**

Microbial diversity analyses showed no significant differences in the overall microbiome after SDF treatment. However, significant changes in the abundance of specific bacteria and fungi, particularly *Lactobacillus* spp., *Bifidobacterium* spp., and *Candida* spp., were observed after treatment. Furthermore, overabundance of *Streptococcus mutans* and *Candida dubliniensis* at baseline was observed in children who had at least one caries lesion not arrested after one SDF application, compared to those who had 100% arrest rates. The overall arrest rates for treated carious lesions were 75.9% at the second visit and 92.8% at the third visit. Arrest rates were higher for all lesions after two applications of SDF with NaF varnish, and applications one month and four months apart had higher arrest rates (95.9% and 98.5%) than six months (81.1%) apart.

**Conclusions:**

Applications of SDF with NaF varnish were an effective modality for arresting ECC, with higher arrest rates after two SDF applications. No loss of diversity but changes in the abundance of specific bacteria and fungi were observed after SDF treatment.

**Trial registration:**

ClinicalTrials.gove NCT04054635 (first registered 13/08/2019).

**Supplementary Information:**

The online version contains supplementary material available at 10.1186/s12903-025-06986-0.

## Introduction

Early childhood caries (ECC), characterized by the presence of tooth decay in primary teeth in children younger than 6 years of age, is a multifactorial disease caused by a combination of factors including oral colonization with increased levels of cariogenic bacteria, decreased microbial diversity, and teeth that are susceptible due to enamel defects (e.g., hypoplasia) and/or frequent consumption of fermentable carbohydrates [1–3]. Within Canada, day surgery for ECC constitutes 31% of all day surgeries for children aged 1–5 years, making this the major cause for day surgeries within this age group [4, 5].

In vitro studies have shown that interkingdom interactions were associated with dysbiosis of the oral microbiome [6, 7]. Both *Candida albicans* and *Streptococcus mutans* develop symbiotic interactions in the presence of sucrose that boost their ability to colonize teeth and synergistically enhance virulence which results in aggressive onset of dental caries lesions [6].

Treatment of young children with severe ECC often requires high-cost general anesthesia and advanced clinical skills which may be unavailable or unaffordable for many pediatric populations [8]. Oral rehabilitation may involve multiple extractions, restorations and crowns [9]. Children treated under general anesthesia often develop new or recurrent caries within months of surgical rehabilitation, with some children requiring subsequent visits to the operating room for dental treatment [10, 11]. Traditional treatment modalities do not address causative factors and have not reduced the incidence of childhood caries and rates for dental surgery in North America [5].

Silver diamine fluoride (SDF) is a simple, non-invasive, alkaline topical fluoride solution containing 38% w/v of Ag(NH_**3**_)_**2**_F. It is commonly used to arrest caries in children. Silver ions affect cariogenic bacteria by deactivating enzymes, mutating or deactivating genetic material, and disrupting cell membranes or cell walls, all of which result in cell death [12]. A solution of 38% SDF (Advantage Arrest, Oral Science, Brossard, Québec, Canada) contains 25% silver, 5.5% fluoride, and 8% ammonia. In the pilot study that preceded our current study, Sihra et al. (2020) concluded that at least two applications of SDF are recommended, with lesion arrest rates of 74.1% and 96.2% after 1 and 2 applications of SDF, respectively [13]. Application of SDF in children too young to tolerate restorative procedures can stabilize active caries until the child is older and able to cooperate for traditional restorative techniques or for minimally invasive techniques [14]. Since treatment with SDF is non-invasive and easily performed, it can be a useful addition to existing traditional treatment modalities [15]. Primary teeth that have been treated with SDF and atraumatic techniques can sustain function and act as a space maintainer until eruption of the permanent successor [15].

Previous studies characterized the effect of SDF treatment on the plaque bacteriome [16, 17], and mechanistic studies investigated the effect of SDF on only few *Candida* spp [18, 19]. However, the influence of the application regimen of SDF on the oral mycobiome is not yet studied. The purpose of this study was to investigate the oral bacteriome and mycobiome changes that result from SDF and sodium fluoride (NaF) varnish applications randomized to three different application frequency regimens. This study is part of a larger randomized clinical trial to investigate the effectiveness of using SDF to arrest caries lesions in children with ECC.

## Methods

The study protocol was approved by the University of Manitoba Biomedical Research Ethics Board (HS24849/B2021:031) and was registered on ClinicalTrials.gov (NCT04054635, first registered 13/08/2019). This study adheres to CONSORT guidelines. Methods for the larger randomized clinical trial have also been published previously [20]. Study visits took place at the Children’s Hospital Research Institute of Manitoba (CHRIM) or at community-based dental clinics in Winnipeg. A total of forty-five children < 72 months of age were initially recruited into this open-label, parallel group, randomized clinical trial based on inclusion and exclusion criteria (Supplementary Table 1) between December 2019 and June 2020 with block randomization to achieve equal proportions in three groups (*n* = 15 per group). Children with active caries lesions (International Caries Detection and Assessment System 5 or 6) in at least one primary tooth that was eligible to receive SDF were included in the study.

Participants were randomized to three different treatment regimens. Prior to recruitment, the research coordinator prepared sealed envelopes with each participant number. The contents of the envelopes were selected randomly and contained details for one of the three regimen types. When a child was recruited into the study, research staff selected the envelope with the appropriate participant number, thus assigning the child to one of three groups. Regimens included application of SDF at a first and second study visit, along with a third follow-up visit (no application of SDF), one month (Regimen 1 M), four months (Regimen 4 M), or six months (Regimen 6 M) apart—at fixed intervals [20]. Regimen 1 M was completed in two months, Regimen 4 M was completed in eight months, and Regimen 6 M was completed in a year. All research personnel, including the examiner, were not blinded to the participant’s regimen group and prior status of lesions.

Supragingival plaque samples were taken on the spot and prior to SDF applications at the first, second, and third study visits. Children were to be excluded if antibiotics were used within the last two weeks. No other specific instructions or restrictions were given to participants prior to sample collection. A sterile interdental brush was scrubbed on all available tooth surfaces [20–22]. Samples were then dislodged into 1 mL of RNAprotect Reagent (Qiagen, Hilden, Germany) and frozen at −80 °C until DNA extraction. Dental plaque samples were taken at first (baseline), second, and third (final) study visits. Cavitated lesions were treated with 38% SDF (Advantage Arrest, Oral Science, Canada) at first and second visits. SDF application was followed by application of 5% NaF varnish (NuPro White, Dentsply Sirona, USA).

Written informed consent, by parents or caregivers, was obtained for each child. Dental exams, assessment of lesions, supragingival plaque collection, and SDF applications were completed by a trained dentist. The color, hardness, and size of lesions, along with dmft (decayed, missing, and filled primary teeth) scores were recorded at each visit [20]. Lesions were deemed arrested if found to be black and hard.

Demographic data, arrest rates, and dmft scores were analyzed using NCSS 2022 statistical software (NCSS, LLC). The data were analyzed using ANOVA, Kruskal-Wallis, Chi-square, Fisher’s exact test, and paired sample *t*-test, when appropriate. *P* < 0.05 was considered significant. With 15 participants per group, our study could achieve a power of 0.91 at a 5% significance level to detect an effect size of 0.4 using repeated measures ANOVA to compare microbial diversity among three study visits.

DNA extraction was completed using the DNeasy PowerSoil Pro Kit (Qiagen, Hilden, Germany). The plaque samples were centrifuged for 10 min at 13,000 rpm. The supernatant was then discarded and 800 µL of buffer CD1 was used to re-suspend the resultant pellet. This mix was then added to the PowerBead Pro Tube and manufacturer’s instructions were followed for DNA extractions.

The library preparation and paired-end Illumina MiSeq PE300 sequencing of the V4 region of bacterial 16 S rRNA and fungal ITS1 (Internal Transcribed Spacer 1) rRNA genes were performed by Genome Quebec Innovation Center. The forward and reverse primers used to amplify the 16 S and ITS1 rRNA genes, respectively, were the V4-16 S 515 F (GTGCCAGCMGCCGCGGTAA) and 806R (GGACTACHVGGGTWTCTAAT), and the ITS1-30 F (GTCCCTGCCCTTTGTACACA) and ITS1-217R (TTTCGCTGCGTTCTTCATCG) [23].

Sequencing data was analyzed using QIIME2 2018.11 (Quantitative Insights Into Microbial Ecology) [22, 24, 25]. DADA2 implemented in QIIME2 was used to filter and merge the pair-end sequences, and to obtain the table of amplicon sequence variants (ASVs) [26]. The ITS1 sequences were trimmed using the Q2-ITSxpress QIIME2 plugin before the DADA2 step [27]. The Human Oral Microbiome Database (HOMD, version 15.1) and the UNITE database (version 8.2; QIIME developer release) were used for the taxonomic assignment of bacteria and fungi, respectively, with classify-sklearn naïve Bayes taxonomy classifier in QIIME2 [21, 25, 28–30]. The fungal ASVs that were assigned only at kingdom level were submitted to further fungal ASV curation with the R package LULU and the program BLASTN in NCBI [31, 32]. The ASVs with non-fungal BLASTN results were discarded and the remaining were repeatedly assigned to new taxonomic assignments using different UNITE databases threshold levels and taxonomy classification methods (q2-feature-classifier classify-sklearn and classify-consensus-blast) in QIIME2 [22, 30, 33–35]. The data was imported into R using the R package “qiime2R” (version 0.99.13) and additional filtering was performed using “phyloseq” (version 1.30.0) to remove singletons [36, 37]. The ASV counts were then normalized using the cumulative-sum scaling (CSS) approach from the R package “metagenomeSeq” version 1.28.2 [38].

The alpha diversity analysis was performed using the Shannon index and raw ASV count data in R (“phyloseq” package, version 1.30.0). Alpha diversity comparisons were done by the Kruskal-Wallis test or Friedman test as appropriate. Beta diversity analysis was performed on CSS normalized ASV data, using weighted UniFrac distances and the permutational analysis of variance (PERMANOVA) test with 999 permutations in the R package “vegan” (adonis function; version 2.5.6) [39]. It was visualized using principle coordinate analysis (PCoA) in the R package “ggplot2” (version 3.3.3) [40].

A paired DESeq2 negative binomial Wald test was used to detect differentially abundant species between groups, controlling the false discovery rate (FDR) for multiple comparison [41]. FDR adjusted *p* < 0.05 was considered significant.

## Results

Forty-five children, 15 children per group, with mean age of 43.5 ± 13.8 months participated. Seventeen (37.8%) were female and 28 (62.2%) were male (Table 1). One child assigned to Regimen 6 M did not return for follow-up, bringing final sample size to 44 participants and 132 plaque samples. No harm or unintended effect was observed.

**Table 1:** Characteristics of study participants

Age at baseline (months), mean ± standard deviation			*p*-value
Total	43.5 ± 13.8		
Regimen 1 M	43.7 ± 14.2		0.09
Regimen 4 M	41.6 ± 12.7		
Regimen 6 M	45.2 ± 15.1		

Sex, ***n*** (%)	Female	Male	*p* -value
Total	17 (37.8)	28 (62.2)	
Regimen 1 M	4 (26.7)	11 (73.3)	0.51
Regimen 4 M	7 (46.7)	8 (53.3)	
Regimen 6 M	6 (40)	9 (60)	

A total of 195 carious lesions in 44 children were treated at baseline and followed over two subsequent study visits. There was a total of 74 teeth treated in Regimen 1 M, 68 in Regimen 4 M, and 53 in Regimen 6 M. One child presented with an abscessed SDF-treated tooth at Visit 3 in Regimen 1 M and this lesion was considered a failure i.e., not arrested. Average arrest rates were higher at Visit 2 and Visit 3 for Regimen 4 M and Regimen 1 M compared to Regimen 6 M (Table 2). Arrest rates were higher for all lesions after two applications of SDF, with a statistically significant increase in the arrest rate from visit 2 to visit 3 for Regimens 4M (*p* = 0.015) and 1M (*p* < 0.001) (Table 2).

**Table 2:** Average dmft scores and arrest rates

	Average dmft			Average arrest rate	
	Visit 1	Visit 2	Visit 3	Visit 2	Visit 3
Regimen 1 M	6.67	6.73	6.73	81.1% (60/74)	95.9% (71/74)
Regimen 4 M	6.40	6.40	6.40	80.9% (55/68)	98.5% (67/68)
Regimen 6 M	6.79	6.93	6.93	62.3% (33/53)	81.1% (43/53)
Total	6.61	6.68	6.68	75.9%* (148/195)	92.8%** (181/195)

^*^Significant difference between Regimens 1 M, 4 M, and 6 M at second visit (Pearson’s Chi-square test, *p* < 0.05)

^**^Significant difference between Regimens 1 M, 4 M, and 6 M at third visit (Pearson’s Chi-square test, *p* < 0.05)

Average dmft score remained the same between visits 1, 2, and 3 for Regimen 4 M. While for Regimens 1 M and 6 M, the average dmft increased between visits 1 and 2 and remained the same at visit 3 (Table 2). The participants did not receive specific instructions to change dietary and oral hygiene habits. No significant changes in frequency of tooth brushing and use of toothpaste with fluoride were observed between baseline and follow-up visits (Supplementary Table 2). Changes in diet were not evaluated.

**Fig. 1:**
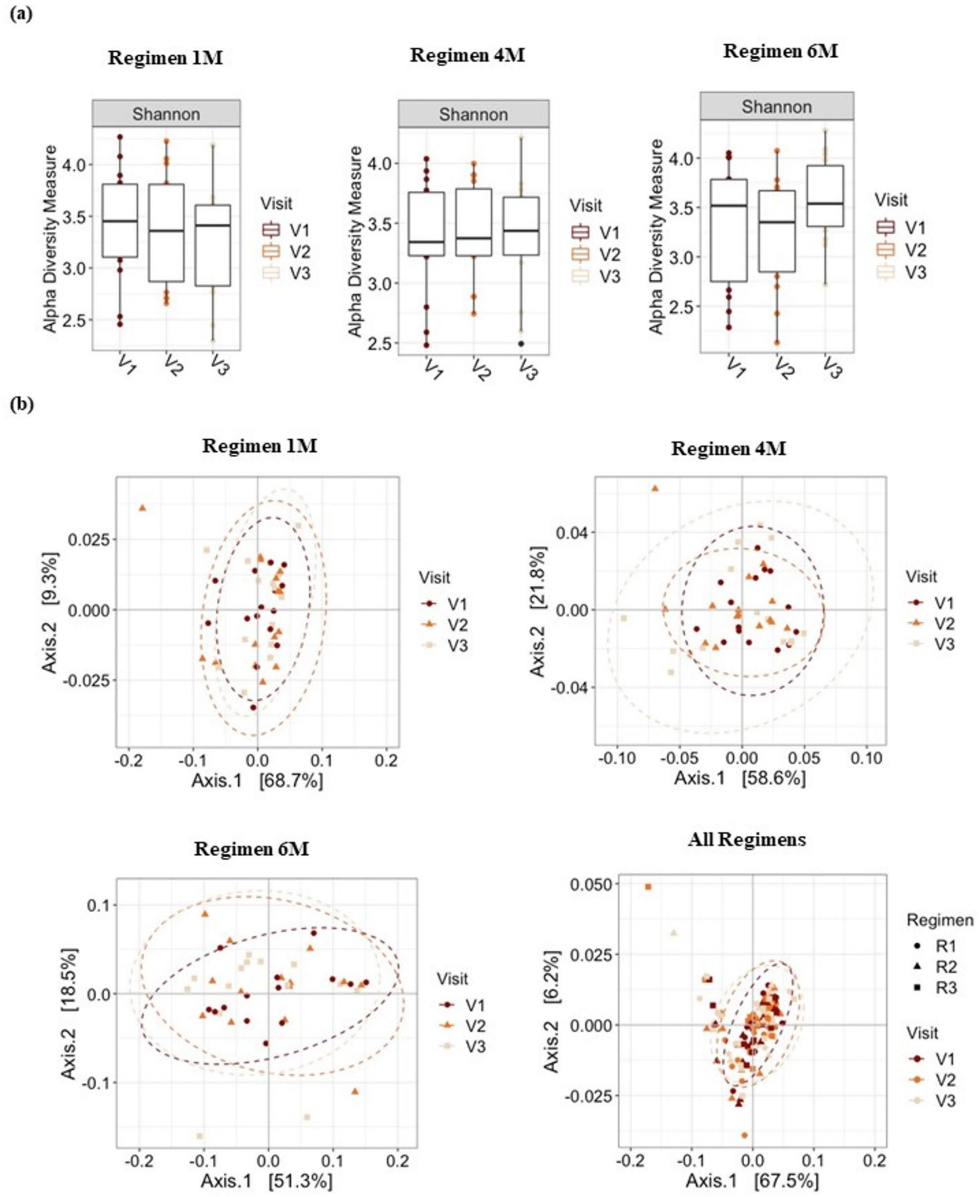
Alpha and beta diversity analyses. **a** Alpha diversity analysis. Boxplot of Shannon Index for bacterial taxa in Regimens 1 M, 4 M, and 6 M, and subgroup analysis by visit. Line inside box represents median. Whiskers represent lowest and highest values within 1.5 interquartile range. **b** Beta diversity analysis. Principal coordinate analysis plots of weighted UniFrac distances based on overall structure of supragingival plaque bacteriome. Each data point represents a sample coloured according to visit. Ellipses represents 95% confidence level. No significant separation of samples was observed between regimens or visits

Alpha diversity analysis showed no significant difference between visits for all regimens, although a slight increase in the average diversity can be observed between visits 2 and 3 (Friedman Test, *p* > 0.05; Fig. 1A). Overall, the beta diversity analysis showed no significant differences in the supragingival plaque bacteriome between visits (Fig. 1B).

**Fig. 2:**
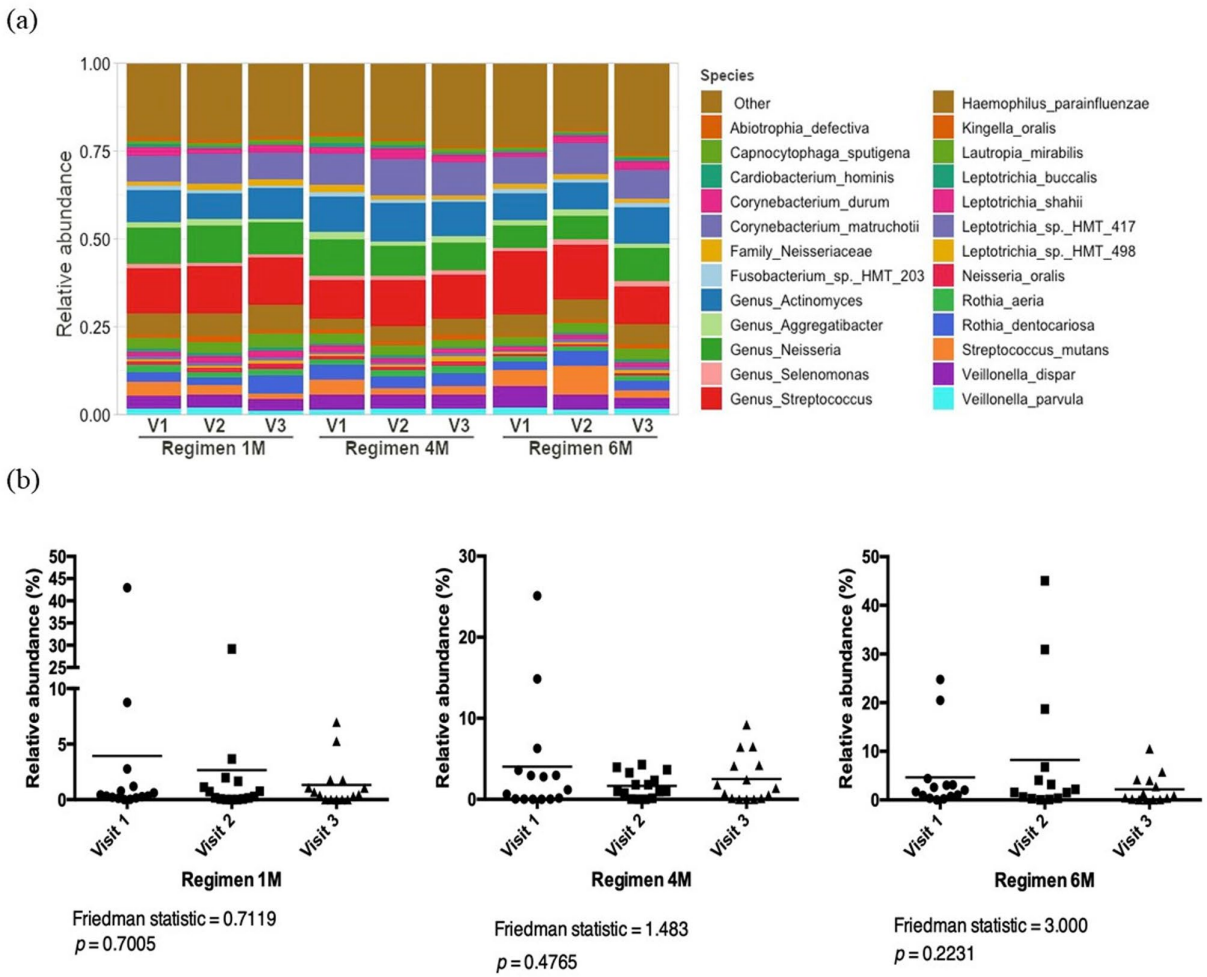
Bacterial taxonomic profile of dental plaque and relative abundance of *Streptococcus mutans*. **a** Taxonomic profiles of dental plaque according to regimens and visit at bacterial species level. Colours assigned only to top 25 most abundant taxa. **b** Relative abundance of *Streptotoccus mutans* according to regimen and visit

Taxonomic assignment showed that *Streptococcus*, *Corynebacterium* and *Actinomyces* were the most abundant genera overall (Fig. 2A). The relative abundance of Streptococcus mutans in different study visits and regimens are shown in Fig. 2B.

**Fig. 3:**
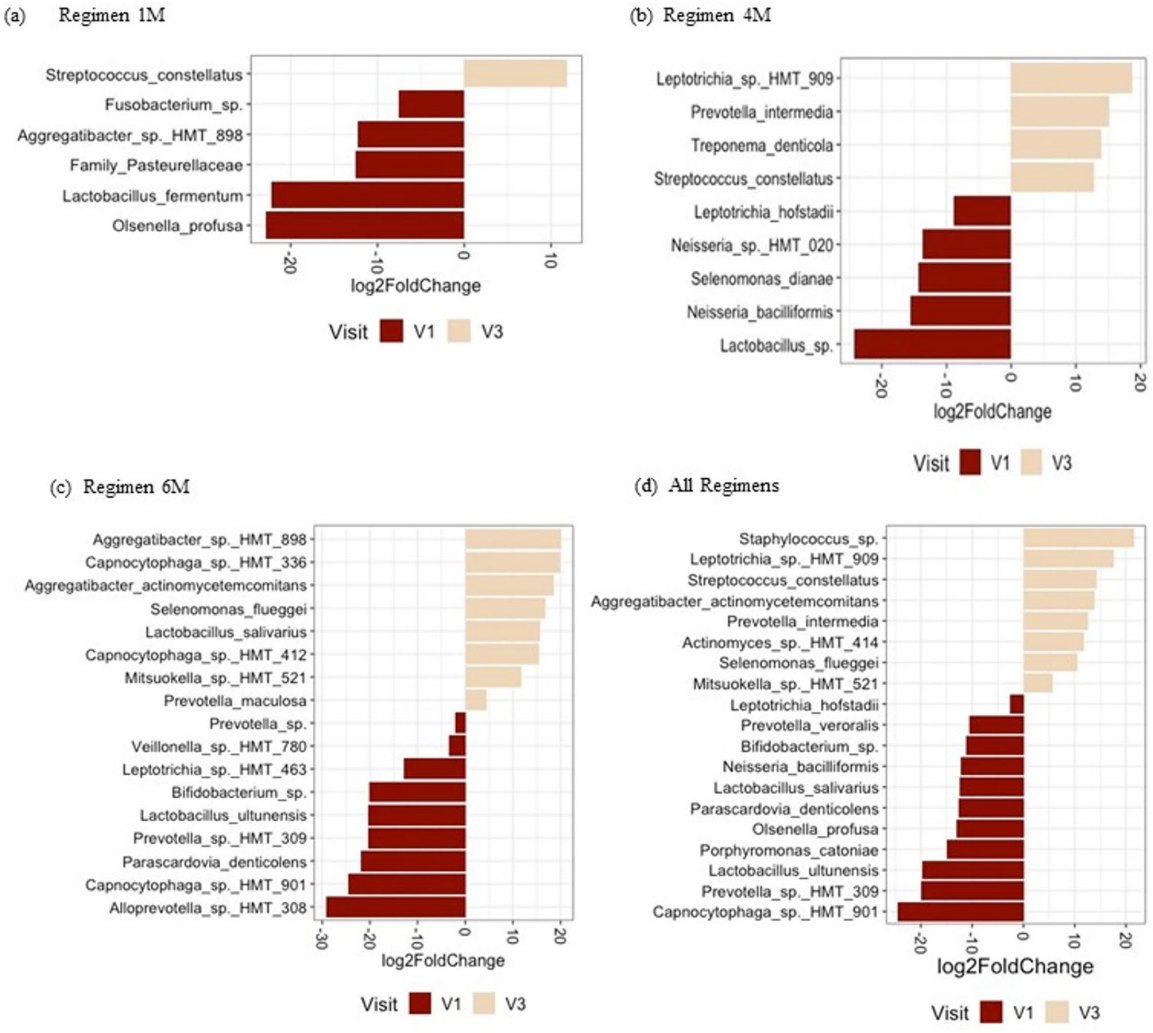
Differential abundance analysis for bacterial species comparing first and third visits. Figure shows relative fold change in bacterial abundance between visits 1 and 3 in (**a**) Regimen 1 M, (**b**) Regimen 4 M, (**c**) Regimen 6 M, and (**d**) all regimens together. Only bacterial taxa with false discovery rate adjusted *p* < 0.05 are shown

The differential abundance analysis showed that various bacterial species were significantly enriched or depleted between visits 1 and 2 or between visits 1 and 3 (Fig. 3 and Supplementary Fig. 2). Compared to the following visits, the supragingival plaque of the children at baseline (visit 1) was enriched with cariogenic bacteria such as *Lactobacillus* spp. and/or *Bifidobacterium* spp. (Supplementary Fig. 2). Children in Regimen 6 M showed higher abundances of Lactobacillus salivarius and higher dmft scores in visits 2 and 3, compared to baseline, and lower arrest rates compared to the other regimens (Fig. 3C, Supplementary Fig. 2E, Table 2).

**Fig. 4:**
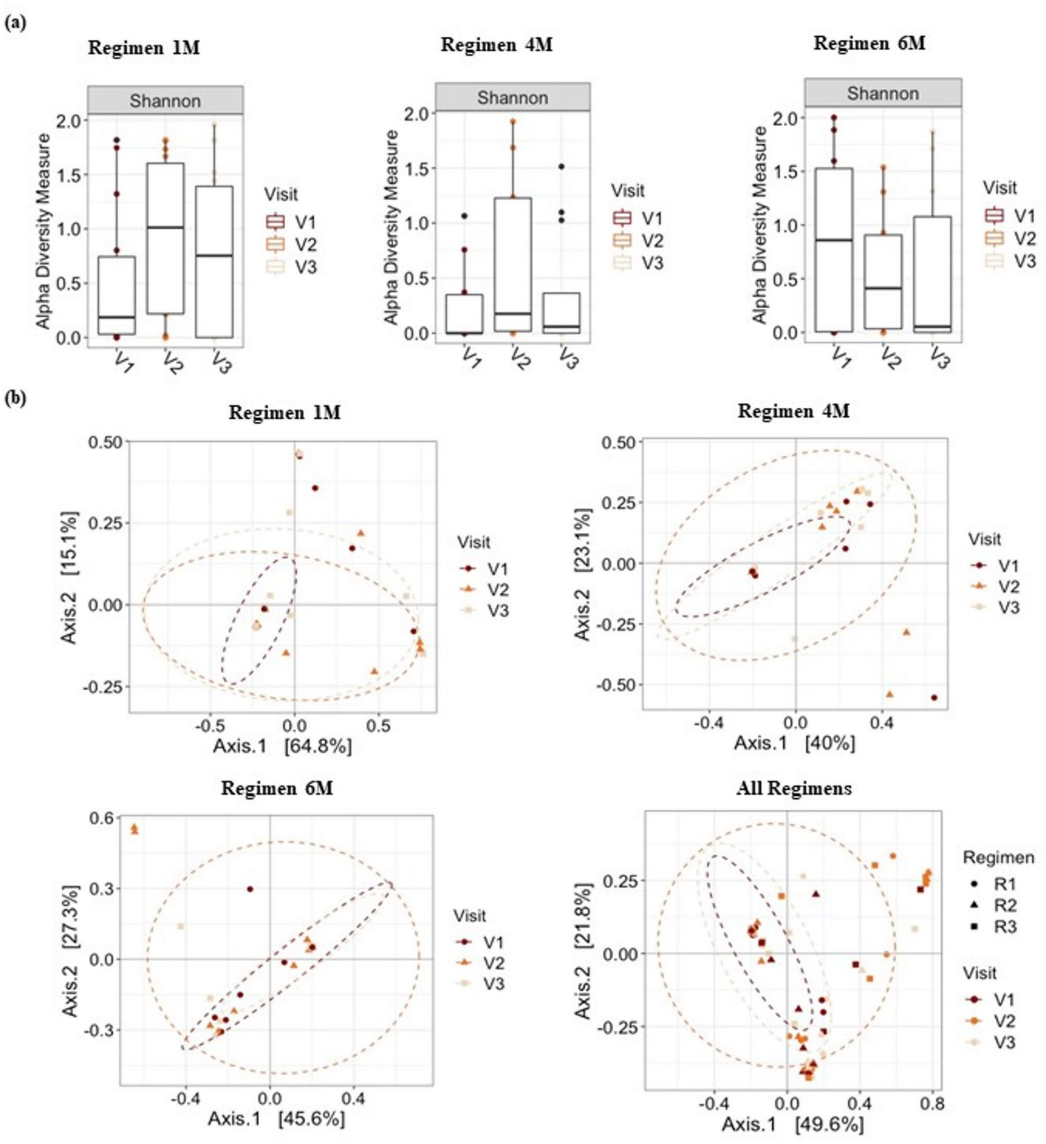
Alpha and beta diversity analyses of fungal microbiome. **a** Alpha diversity analysis. Boxplot of Shannon Index for fungal taxa in Regimens 1 M, 4 M, and 6 M, and subgroup analysis by visit. Line inside box represents median. Whiskers represent lowest and highest values within 1.5 interquartile range. **b** Beta diversity analysis of fungal communities. Principal coordinate analysis plots of weighted UniFrac distances based on overall structure of supragingival plaque mycobiome. Each data point represents a sample coloured according to visit. Ellipses represent 95% confidence level. No significant separation of samples was noted between regimens or visits

Five samples did not pass quality control filtering steps for the mycobiome data analysis and, due to the design of the study, 18 samples were removed to allow for paired analysis of the remaining 114 plaque samples. The alpha diversity analysis showed no significant differences between visits for all groups (Friedman Test, *p* > 0.05; Fig. 4A). Similarly, the beta diversity analysis showed no significant difference in supragingival plaque mycobiome between visits for all groups (Fig. 4B).

**Fig. 5:**
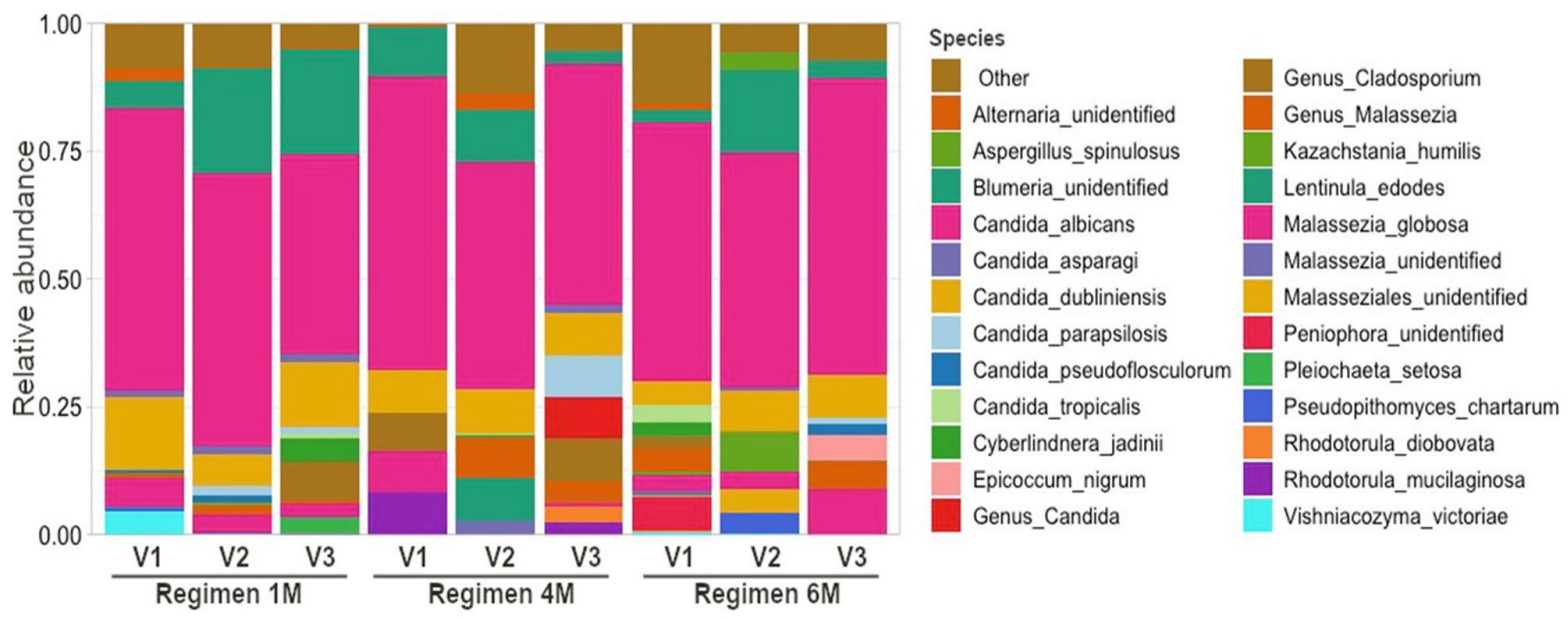
Taxonomic profiles of children’s dental plaque according to regimen and visit at fungal species level. Colours were assigned only to the top 25 most abundant taxa

Taxonomic assignment showed that *Candida*, *Blumeria*, and *Malassezia* were the most abundant genera. Fig. 5 shows the relative abundances of the top 25 most abundant fungal taxa and it shows that *Candida albicans* was highly abundant in all groups regardless of number of visits or regimens.

**Fig. 6:**
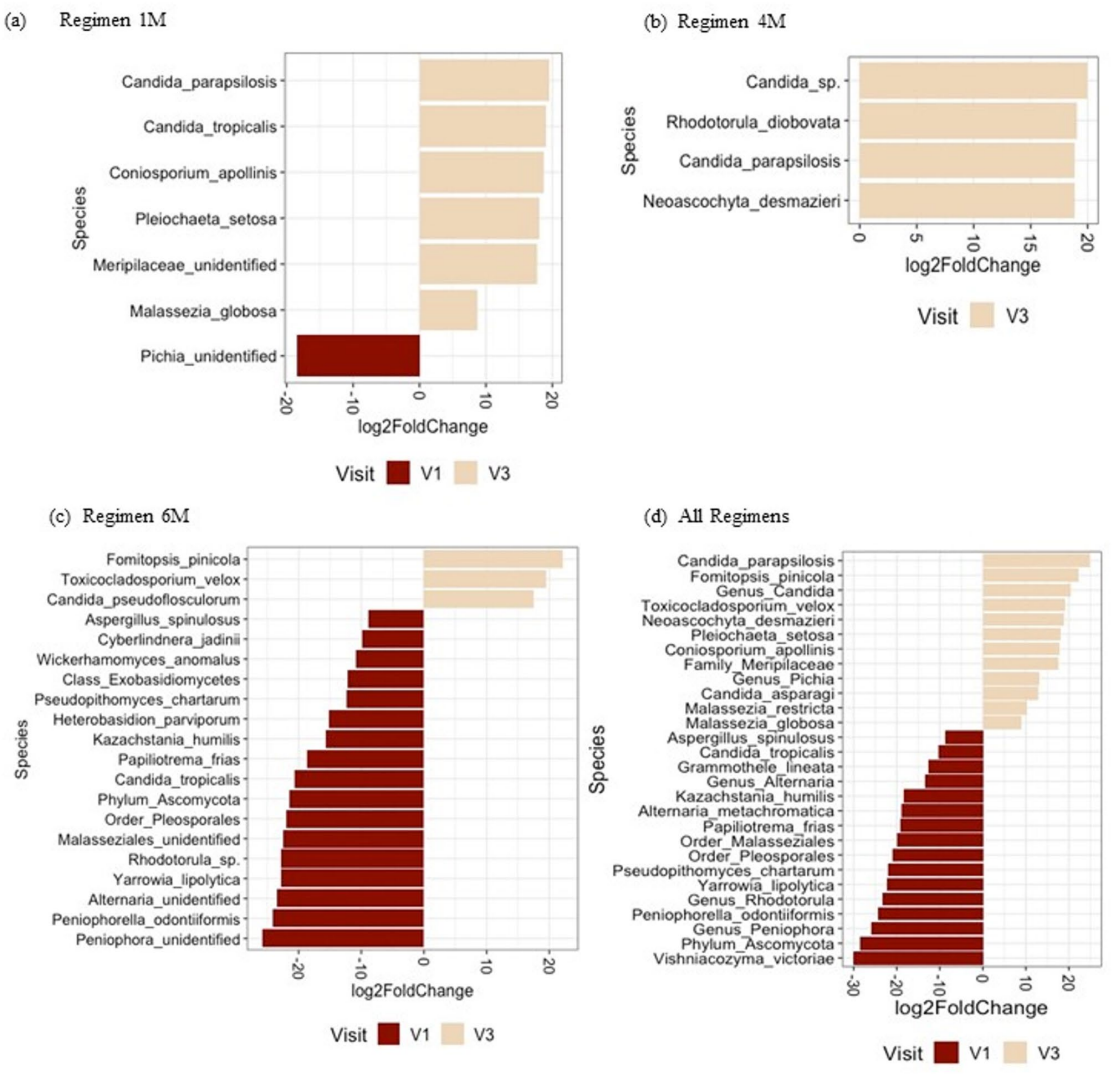
Differential abundance analysis for fungal species comparing visits 1 and 3. Figure shows relative fold change in fungal abundance between visits 1 and 3 in (**a**) Regimen 1 M, (**b**) Regimen 4 M, (**c**) Regimen 6 M, and (**d**) all regimens together. Only fungal taxa with false discovery rate adjusted *p* < 0.05 are shown

The differential abundance analysis showed many fungal species that were significantly enriched or depleted between visits in all regimens (Fig. 6, Supplementary Figs. 3 and 4). A significant decrease in Candida tropicalis was noted from visit 1 to visit 3 in Regimen 6 M. This decrease was also seen from visit 2 to visit 3 and visit 1 to visit 3 when all regimens were taken together. All these results taken together suggest that SDF treatment may have an effect on the abundance of specific fungi but it does not modify the overall microbial structure of the supragingival plaque.

**Fig. 7:**
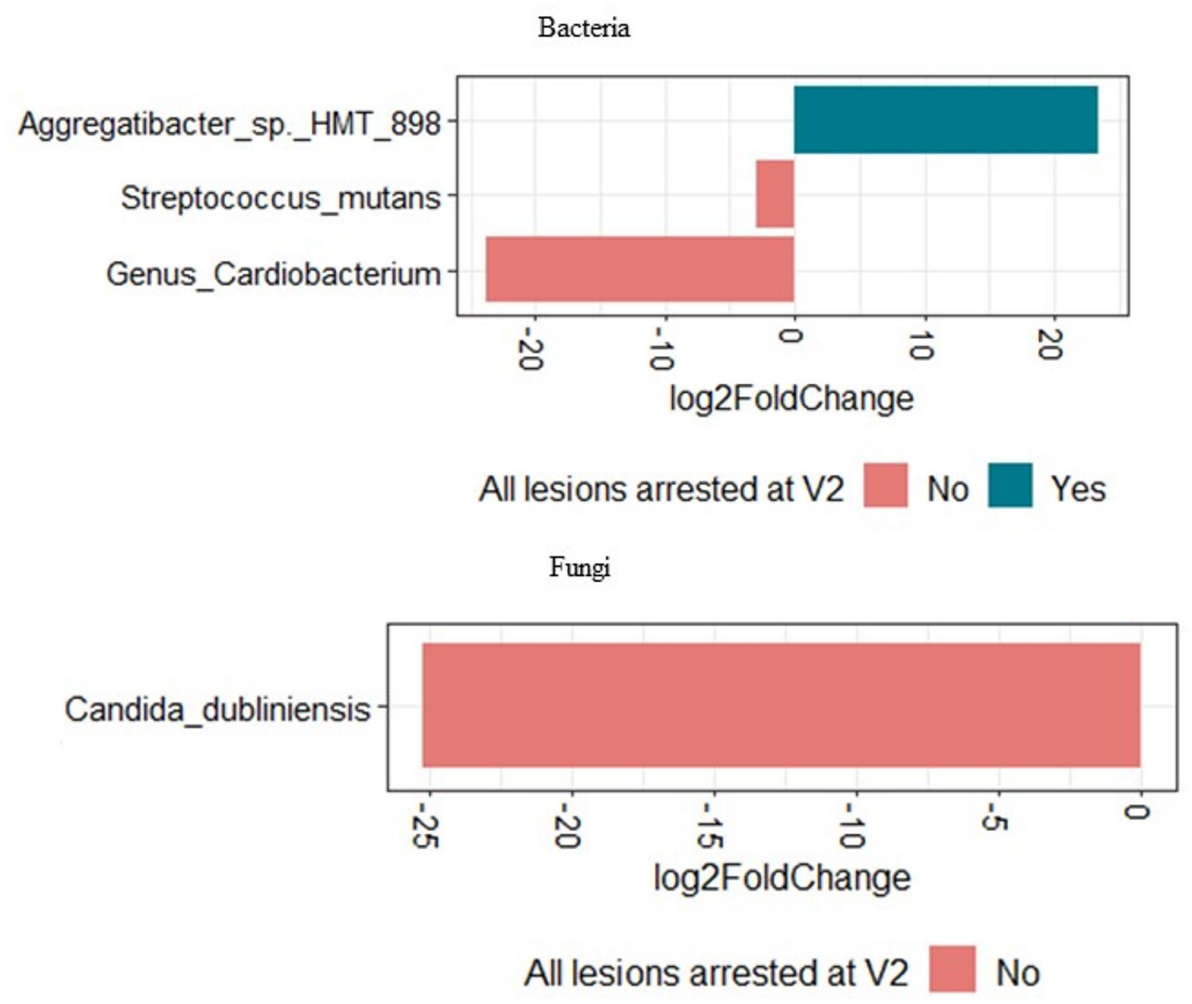
Differential abundance analysis. Comparing differences in baseline microbiome between children with 100% lesions arrested (yes) and those with < 100% arrest rates (no) after one SDF application. Only baseline (before SDF treatment) microbiome data was used for analysis

After only one application of SDF, 40.9% (*n* = 18/44) of the children had 100% of the treated caries lesions arrested. While, 59.1% (*n* = 26/44) of the children had at least one lesion that was not arrested at visit 2. Among those children, six had anterior lesions not arrested, 16 had posterior lesions not arrested, and four had both anterior and posterior lesions not arrested. To evaluate if the baseline microbiome is linked to SDF treatment outcomes, we compared the baseline microbiome of children who had 100% of caries arrested after one application of SDF and those with at least one lesion not arrested after one application of SDF (i.e., at visit 2). For this we used differential abundance analysis adjusting for differences in the regimen used and the sex of the child. Interestingly, the dental plaque of the children with lesions not arrested after one SDF application was enriched with the caries associated species *Streptococcus mutans* and *Candida dubliniensis* at baseline, compared with children who had 100% arrest rates (Fig. 7).

## Discussion

SDF treatment in children too young to tolerate restorative procedures can stabilize active caries until the child is older and able to cooperate for traditional restorative or minimally invasive techniques [14]. In a pilot study, it was concluded that at least two applications of SDF are recommended, with 96.2% of arrest rate achieved, which is similar to our findings [13]. Since SDF treatment is non-invasive and easily performed, it can be a useful addition to existing traditional treatment modalities [15]. Primary teeth that have been treated with SDF and atraumatic techniques can sustain function and act as a space maintainer until eruption of the permanent successor [15]. The recruitment of children from local community dental clinics provides a good representation of the targeted pediatric population in need of SDF treatment for dental caries [20]. The specificity of the eligibility criteria also helps mitigate sampling bias.

The current AAPD Clinical Practice Guideline recommends monitoring carious lesions for arrest at 2–4 weeks following application of SDF with reapplication as necessary to achieve arrest of all targeted lesions and re-care monitoring based on disease activity and caries risk level at three, four, or six months [42]. SDF is known to have a reservoir effect with sustained antimicrobial effects over time [43]. Application to carious lesions is as effective in preventing caries in other teeth and surfaces as applying SDF directly [43]. In our study, the average dmft remained the same over all three visits for Regimen 4 M which is consistent with these findings. Average arrest rates were higher for all groups after two applications of SDF followed by NaF varnish, compared to one application of SDF followed by NaF varnish, however, arrest rates were higher with application frequencies of 1 month and 4 months, compared to 6 months.

Regimen 6 M showed lower arrest rates compared to Regimen 1 M and 4 M and also showed increased dmft between visit 1 and visits 2 and 3. The literature suggests that increasing the frequency of SDF application can increase caries arrest rates [42]. The effectiveness of lesion arrest by 38% SDF decreases over time [42, 44]. Re-activation of treated lesions has been observed in a two-year study where a single application of 38% SDF was sufficient to prevent only 50% of the arrested surfaces at six months from reverting to active lesions again over 24 months [44]. We suggest that six months between applications may not be optimal for arrest of caries in high-risk children. Other factors which could be influencing arrest rates and dmft with six months application frequency and that need to be investigated in future studies include the number of erupted teeth, diet high in fermentable carbohydrates, plaque index, amount of plaque present in lesions, medical status, and use of antibiotics.

Previous studies have looked at microbial changes following SDF treatment in pediatric patients [16, 45]. They looked at changes in the bacteriome at the caries lesions only and had smaller sample sizes. Milgrom et al. (2018) found no consistent changes in relative abundance of caries-associated microbes or lesion-specific microbial diversity before treatment and at follow up (14–21 days) for three children from each group (38% SDF vs. placebo) [45]. In our study, no significant differences in alpha or beta diversity were observed in the overall plaque microbiome after SDF treatment. This suggests that SDF and NaF varnish treatments may not change the overall microbial diversity of the supragingival plaque. However, as plaque samples were collected from all available tooth surfaces, it is not possible to determine if there were localized changes in the microbiome.

Mei et al. (2020) found no overall microbiome changes in five-year-old children immediately before and weeks after one application of 38% SDF [16]. Microbiota showed a temporal shift in the positive direction after two weeks but returned to original status after twelve weeks. The authors noted that *Streptococcus mutans* tended to decline in arrested carious lesions while *Neisseria* species and *Actinomyces naeslundii* tended to increase in arrested carious lesions. Carious lesions that remained active after SDF treatment showed a trend of increased abundance of *Streptococcus mutans*,* Streptococcus sobrinus*, and *Lactobacillus* spp. compared to pre-SDF treatment levels, though not all were statistically significant. Takahashi et al. (2021) showed that 38% SDF can decrease the number of *Streptococcus mutans*, along with the amount of water-insoluble glucan, and the thickness of the formed biofilm in vitro [46]. They suggest that this may be partially due to an inhibitory effect of silver ions on glucosyltransferase activity (which is used by *Streptococcus mutans* to synthesize extracellular polysaccharides from sucrose) and the rupture of the bacterial cells. This may explain why the supragingival plaque bacteriome showed a decrease in relative abundance from visit 1 to visit 3 for *Streptococcus mutans* for all regimens. However, this trend was not found to be statistically significant (Fig. 2B).

Beyond direct antimicrobial effects, SDF and fluoride varnish provides surface protection [12]. Silver ions, from SDF, can interact with collagen and form a silver-protein complex that indirectly protects dentin collagen by inhibiting enzymes that break down the protein, such as matrix metalloproteinases and cysteine cathepsins [47, 48]. Fluoride, from SDF and NaF varnish, can produce fluorapatite and calcium fluoride which play a role in protecting and re-mineralizing the tooth structure (12). Thus, it is possible that changes in the abundance of caries-associated microbial species could also be driven by ecological changes in the oral environment.

It was also noted that the plaque microbiome was depleted of other cariogenic bacteria and *Candida* species after two applications of SDF. For instance, when all regimens were taken together, *Lactobacillus salivarius* was enriched in visit 1 compared to visit 2 and visit 3, signifying a decrease in abundance from visit 1 to visit 3. *Lactobacillus salivarius* is known to be a dominant species commonly isolated from the dentition of adults and children with caries [49].

High levels of *Candida albicans* are frequently detected in the plaque biofilms of children with ECC [50–52]. Other *Candida* species, such as *Candida tropicalis*, *Candida krusei*, and *Candida glabrata*, are also detected in smaller quantities in the plaque biofilms of children with ECC [50]. In our previous study on the characterization of the oral microbiome in children with S-ECC, we found that *Candida dubliniensis* and *Candida tropicalis* were more abundant in oral swab samples of children with S-ECC compared to caries-free controls [22]. In contrast, healthy, ECC-free children have plaque biofilms where *Candida albicans* is absent or sporadically detected [50–52]. *Candida albicans* was found to be highly abundant in all groups regardless of the number of visits or regimens. Our differential abundance analysis showed many fungal species that were significantly enriched or depleted between visits 1 and 2 or between visits 1 and 3 suggesting that SDF and NaF varnish treatment may have an effect on the abundance of specific fungi.

A study by Fakhruddin et al. (2020) has shown that SDF has antifungal effects against some Candida species including *Candida albicans*,* Candida krusei*,* Candida tropicalis* and *Candida glabrata* [18]. It is noteworthy that some of the fungal and bacterial DNA identified in this study could represent transient colonizers originating from food intake and/or mouth breathing and may not represent the core oral microbiome of preschool children. Future studies are needed to investigate the role of those microorganisms in the oral health of pre-school children.

Although the same number of children were recruited for each group, the total number of teeth treated per group varied, with the Regimen 1 M having the highest number of teeth treated (74) and the Regimen 6 M having the lowest number of teeth treated [53], due to the loss of one participant in Regimen 6 M. There was no intra-examiner analysis done to support the reliability of the clinical records made—this is a limitation for this study. Moreover, dietary habits, quality of the oral hygiene, and presence of visible plaque, may lead to ecological changes in the habitat and influence the outcome of SDF treatment and oral microbiome. For instance, it has been shown that caries lesions with visible plaque are less likely to arrest than those without visible plaque [53]. Therefore, these factors should be taken into consideration in future studies related to SDF treatment. In this study, plaque samples were not site-specific. Thus, data on the microbiome could not be localized to the site of the carious lesion. Comparing overall microbiome changes with site-specific changes would help determine whether SDF has a more pronounced local effect than overall oral microbiome effect. Most oral fungi are present at low biomass and may be difficult to detect in oral samples [54]. In our study, five samples had low number of sequence reads and a total of eighteen samples had to be removed to allow for paired analysis. As the study focused on ECC and included only children younger than 6 years of age, our findings cannot be generalized for all age groups.

## Conclusions

This study provided information on the effect of SDF treatment on the overall dental plaque microbiome of young children with ECC. No significant loss of microbial diversity was seen after SDF treatment. Moreover, significant changes in the abundance of bacterial and fungal species, particularly caries-associated species, were identified. Further studies with a larger sample size are needed to confirm whether the presence or absence of various bacterial and fungal species are the result of SDF applications at various frequencies. SDF with NaF varnish was an effective modality for arresting caries with higher arrest rates for all lesions after two applications. Applications one month or four months apart had higher arrest rates than applications six months apart—this interval may not be optimal for arresting caries in high-risk children.

## Supplementary Information

Supplementary Material 1.

## Data Availability

De-identified raw sequence reads will be publicly available at the NCBI Sequence Read Archive (SRA) Repository as of the date of publication (BioProject accession number PRJNA1085849). Further enquiries can be directed to the corresponding author.

## Abbreviations

ASV: Amplicon sequence variants
CHRIM: Children’s Hospital Research Institute of Manitoba
ECC: Early childhood caries
FDR: False discovery rate
NaF: Sodium fluoride
QIIME: Quantitative Insights Into Microbial Ecology
SDF: Silver diamine fluoride
