# Supplementary material for: Effects of silver diamine fluoride on oral bacteriome and mycobiome: a randomized clinical trial
Source: BMC Oral Health. 2025 Oct 21;25:1643. doi: 10.1186/s12903-025-06986-0 (PMC12539150; doi:10.1186/s12903-025-06986-0)
Supplement: Supplementary file 1 — Supplementary Material 1. [file 12903_2025_6986_MOESM1_ESM.docx]

Supplementary Table 1. Inclusion and exclusion criteria

| Inclusion criteria | Exclusion criteria |
| --- | --- |
| - Child is under 72 months of age with ECC with active caries lesions. - Child has ≥ 1 primary tooth with caries that is eligible to receive silver diamine fluoride. - Eligible primary teeth must have soft cavitated caries lesions extending into dentin (International Caries Detection and Assessment System [ICDAS] 5 or 6). - The cavitated lesions must allow for direct application of silver diamine fluoride. | - Child is allergic or has sensitivity to silver. - Child has hereditary generalized developmental defects of enamel (e.g., amelogenesis imperfecta and dentinogenesis imperfecta). - Child has severe medical problems that limit participation. - Child requires immediate rehabilitation under general anesthesia because of severe infection or pain. - Antibiotic use within the last two weeks. - Any teeth that meet PUFA (pulpal involvement, ulceration due to trauma, fistula, abscess) criteria. The child may still qualify if other eligible teeth with caries do not meet PUFA criteria. |

Supplementary Table 2. Oral hygiene habits

|  | Visit 1 | Visit 2 | Visit 3 | *p*-value  V1xV2 | *p*-value  V1xV3 |
| --- | --- | --- | --- | --- | --- |
| Frequency of toothbrushing | | | | | |
| Regimen 1M (*n* = 15) | | | | | |
| 2x/day | 9 (60%) | 10 (66.7%) | 9 (60%) | 1 | 1 |
| Less than 2x/day | 6 (40%) | 5 (33.3%) | 6 (40%) |  |  |
| Regimen 4M (*n* = 15) | | | | | |
| 2x/day | 7 (46.7%) | 9 (60%) | 7 (46.7%) | 0.7 | 1 |
| Less than 2x/day | 8 (53.3%) | 6 (40%) | 8 (53.3%) |  |  |
| Regimen 6M (*n* = 14) | | | | | |
| 2x/day | 8 (57.1%) | 9 (64.3%) | 11 (78.6%) | 1 | 0.4 |
| Less than 2x/day | 6 (42.9%) | 5 (35.7%) | 3 (21.4%) |  |  |
| Use of toothpaste with fluoride | | | | | |
| Regimen 1M (*n* = 14) | | | | | |
| Yes | 12 (80%) | 13 (86.7%) | 12 (80%) | 1 | 1 |
| No/unsure | 3 (20%) | 2 (13.3%) | 2 (13.3%) |  |  |
| Regimen 4M (*n* = 15) | | | | | |
| Yes | 14 (93.3%) | 12 (80%) | 13 (86.7%) | 0.6 | 1 |
| No/unsure | 1 (6.7%) | 3 (20%) | 2 (13.3%) |  |  |
| Regimen 6M (*n* = 14) | | | | | |
| Yes | 10 (71.4%) | 14 (100%) | 13 (92.9%) | 0.1 | 0.3 |
| No/unsure | 4 (28.6%) | 0 (0%) | 1 (7.1%) |  |  |

**Second visit:**

- Follow-up questionnaire.
- Dental examination (assess lesions).
- Dental plaque samples taken.
- Second application of 38% silver diamine fluoride.

**Second visit:**

- Follow-up questionnaire.
- Dental examination (assess lesions).
- Dental plaque samples taken.
- Second application of 38% silver diamine fluoride.

**Second visit:**

- One participant lost to follow-up (*n* = 1).
- Follow-up questionnaire.
- Dental examination (assess lesions).
- Dental plaque samples taken.
- Second application of 38% silver diamine fluoride.

**Third visit:**

- Final questionnaire.
- Dental examination (assess lesions).
- Dental plaque samples taken.

**Third visit:**

- Final questionnaire.
- Dental examination (assess lesions).
- Dental plaque samples taken.

**Third visit:**

- Final questionnaire.
- Dental examination (assess lesions).
- Dental plaque samples taken.

**One-month interval group**

(*n* = 15)

**Six-month interval group**

(*n* = 15)

**Four-month interval group**

(*n* = 15)

**First visit:**

- Baseline questionnaire.
- Dental examination.
- Dental plaque samples taken.
- First application of 38% silver diamine fluoride.

**First visit:**

- Baseline questionnaire.
- Dental examination.
- Dental plaque samples taken.
- First application of 38% silver diamine fluoride.

**First visit:**

- Baseline questionnaire.
- Dental examination.
- Dental plaque samples taken.
- First application of 38% silver diamine fluoride.

**Enrolment**

- Children with early childhood caries recruited from community dental clinics in Winnipeg from December 2019 to June 2020.
- Parents/caregivers provide written informed consent.

**Randomized** (*n = 45)*

One month later

Four months later

Six months later

Six months later

Four months later

One month later

Analysis (*n* = 15)

Analysis (*n* = 15)

Analysis (*n* = 14)

**Supplementary Figure 1. Flow diagram.** Depicts study process (recruitment, randomization, visits and activities, duration, and analysis).

**Supplementary Figure 2. Differential abundance analysis for bacterial species.** Regimen (a-b) 1M, (c-d) 4M, (e-f) 6M, and (g-h) All regimens together. The figure shows the relative fold change in bacterial abundance between (a, c, e, and g) visits 1 and 2 and (b, d, f, and h) visits 2 and 3. Only bacterial taxa with FDR adjusted *p* < 0.05 are shown.


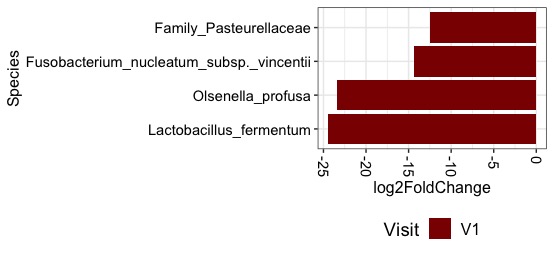


(a) Regimen 1M, V1 vs. V2

(b) Regimen 1M, V2 vs. V3


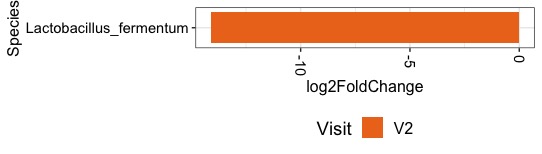

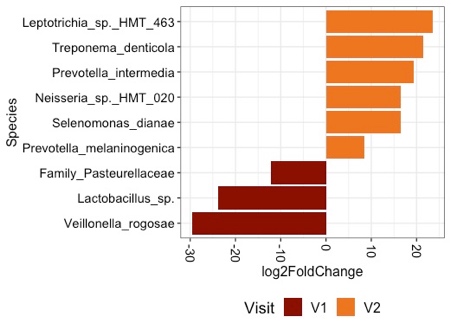


1. Regimen 4M, V1 vs. V2

(d) Regimen 4M, V2 vs. V3


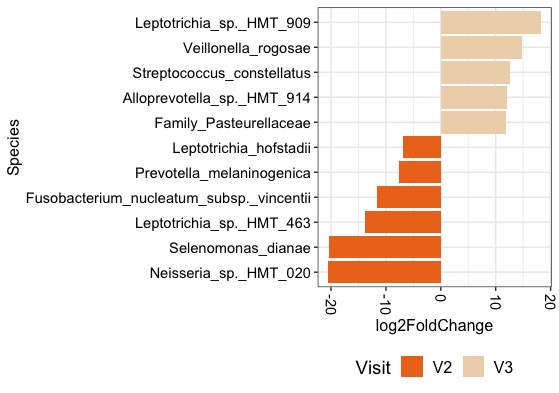

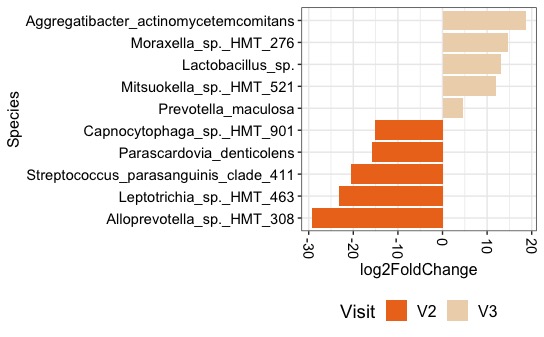

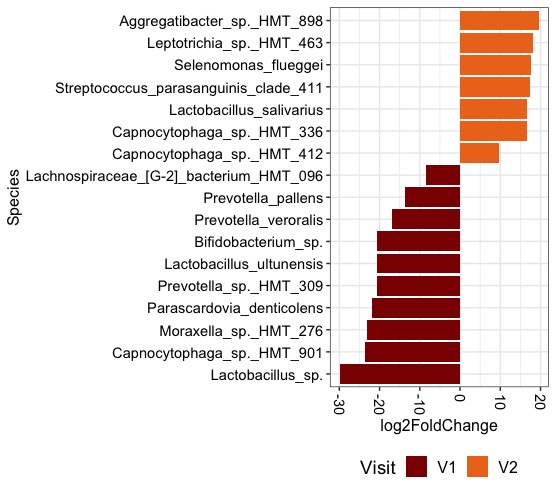


1. Regimen 6M, V1 vs. V2

(f) Regimen 6M, V2 vs. V3


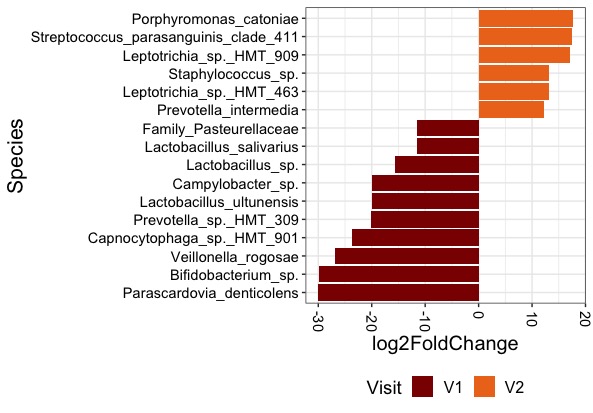


(g) All Regimens, V1 vs. V2

(h) All Regimens, V2 vs. V3


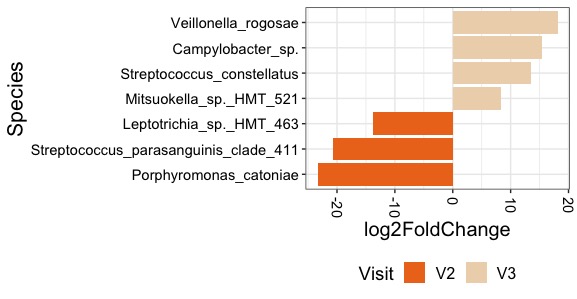

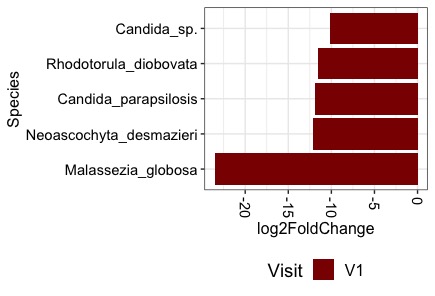


1. Regimen 4M, V1 vs. V2

(d) Regimen 4M, V2 vs. V3


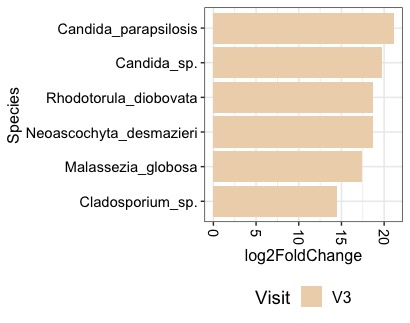

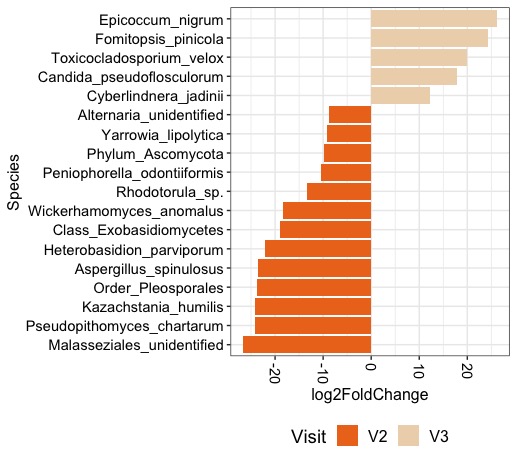

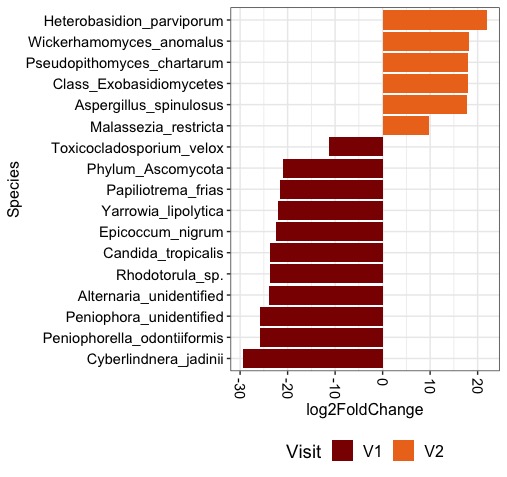


1. Regimen 6M, V1 vs. V2

(f) Regimen 6M, V2 vs. V3


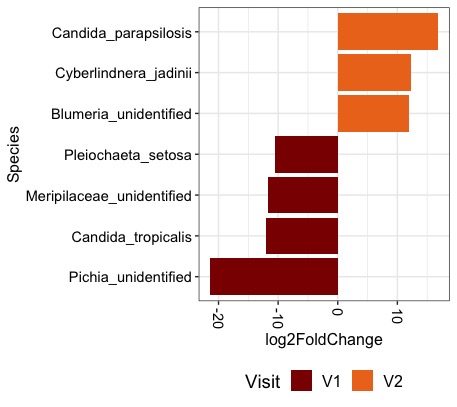


(a) Regimen 1M, V1 vs. V2

(b) Regimen 1M, V2 vs. V3


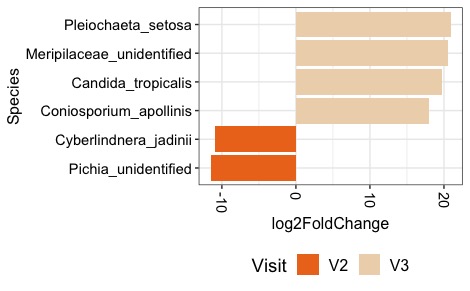


**Supplementary Figure 3. Differential abundance analysis for fungal species.** Regimen (a-b) 1M, (c-d) 4M, and (e-f) 6M. The figure shows the relative fold change in fungal abundance between (a, c, and e) visits 1 and 2 and (b, d, and f) visits 2 and 3. Only fungal taxa with FDR adjusted *p* < 0.05 are shown.


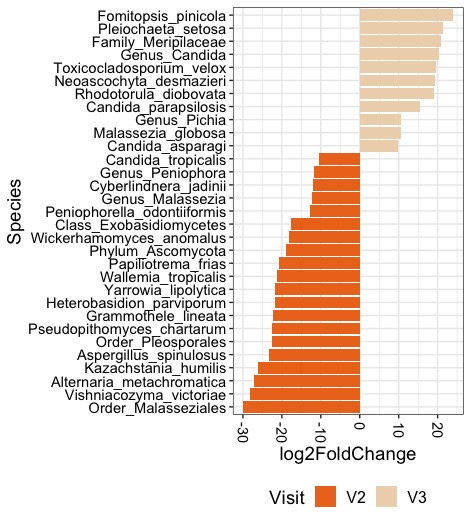

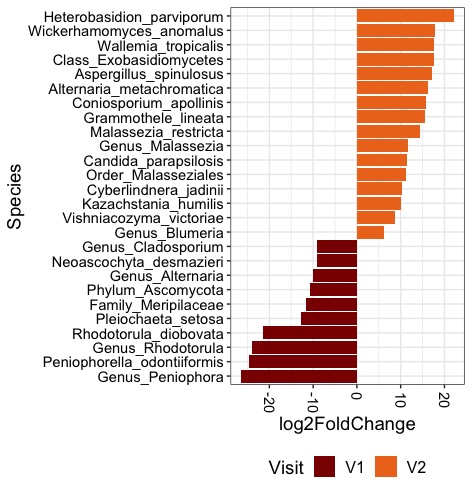


1. All Regimens, V1 vs. V2

(b) All Regimens, V2 vs. V3

**Supplementary Figure 4. Differential abundance analysis for fungal species in all regimens together.** The figure shows the relative fold change in bacterial abundance between (a, c, e, and g) visits 1 and 2 and (b, d, f, and h) visits 2 and 3. Only fungal taxa with FDR adjusted *p* < 0.05 are shown.
